# One Way of Increasing a Dentist’s Usefulness

**Published:** 1889-12

**Authors:** William D. Kempton

**Affiliations:** Cincinnati


### One Way of Increasing a Dentist's Usefulness.

BY WILLIAM D. KEMPTON, M D , D.D.S , CINCINNATI.

Read before the Ohio State Dental Society, Cleveland, October, 1889.

In this age of electric motors and natural gas, the demands on the knowledge, the skill and the endurance of the dental surgeon are daily increasing, and how best to meet this demand is a problem that is commanding the attention of many of our profession. The solutions have been many and various. With some it consists in a thorough knowledge of the nature and causes of decay, and in their enthusiasm they have pursued their studies and researches so far, and have become so familiar with the little bugs which infest our mouths that they are able to give us an accurate description of their manners and customs, and their relationships down to cousins of the forty-second degree. With others it is the discovery of the best material for replacing lost tooth structure, and they grow eloquent in advocating this or that material to the exclusion of all others. With still others it is the best method of introducing these materials, and they tell us, with an air that disarms our doubts, of their ability to drill out and fill nerve canals in comparison with which the most torturous ram's horn is remarkably straight. With others, again, it is the use of the latest and most improved machinery, and they have devised apparatus whose shapes are wonderful, whose mechanism is intricate, and whose uses, in some cases, a mystery to God. None of these solutions, however, take into consideration the dentist himself who may possess all this knowledge and ability, and yet be like an ocean grayhound without steam, or a watch that is unwound.

This generation, with its increasing wealth, its high pressure system of education and the fierce struggle of many to keep up appearances, is bringing to the dentist's care an annually increasing number of pampered children, hysterical girls and excitable women to whom the slightest suggestion of pain brings visions of the Spanish Inquisition in all its horrible details, and who are seized with convulsions at the sight of an instrument and shrink from the contact of even an innocent piece of cotton.

To the conscientious dentist who desires to be as thorough as possible and to avoid inflicting pain, the tears, the piteous appeals for mercy, the restlessness of such patients, the continual seizing of his hands, the frequent and causeless starts, and their repeatedly begging him to desist, all tend to use up, wear out and exhaust his reserve supply of nerve force, and make him nervous, irritable, cranky and impatient, and unfits him for the proper discharge of his duties.

The teeth of these persons, however, continue to decay and to need attention, and as the financial condition of the average dentist will not admit of his turning such patients away, something must be done to brace up his weakened nervous system. On this point I think we will all agree, but when it comes to prescribing a remedy our views widely differ. Some, thinking that all they needed was exercise, have gone into their laboratories to swing dumb-bells and to brandish Indian clubs to the great jeopardy of the shelving. They have done this, probably a week, possibly a month, but rarely longer, when on failing to experience the expected improvement they have given up in disgust. Others again have tried walking, only to find that they were still further exhausted and less fit for duty. One cause of the failure of these remedies lies in the fact that they are resorted to solely as curative measures, just as we would take a dose of epsom salts, and for that reason soon become tiresome. Any remedy, therefore, to be efficacious must combine exercise and recreation with fresh air and sunshine; and no one thing does this to the same degree that the bicycle does. It is because I speak from experience that I make this assertion so positively. Six years ago my appetite was poor and headaches were common, and neither walking nor dumb-bells afforded me any relief. In despair I bought a bicycle, and, after learning to ride it, soon found that I ate more, took less medicine, and felt better than I had for years. In suggesting this remedy I am aware that it will meet with several objections. Some say that bicycles are dangerous. Well, perhaps they are, but so are bath tubs, for people have been drowned in them, yet no sane person would urge that as an excuse for uncleanliness. Ninety-nine per cent. of the accidents to wheelmen are due either to recklessness or carelessness and are avoidable. In six year's experience I have never met with an accident that in any way incapacitated me for practicing my profession. Bicycles, in fact, are much less dangerous than horses, for they neither bite, run away, nor kick.

It is also said that it is a difficult matter to learn to ride. This point is as well taken as the previous one. I have known persons, in charge of a good rider, to be fully a half hour in learning to ride. For that reason, alone there are only a half million cycles in use in England, while in this country the League of American Wheelmen, which includes less than one-tenth of our riders, has only about twelve thousand members.

Again, it is said that bicycle riding is only indulged in by the young and frivolous, and is therefore not just the thing for the dignified dental practitioner. True again. I have never heard of more than two persons learning to ride at the age of seventy-five years. Then the Cincinnati Bicycle Club, to which I belong, has only two members that are past fifty years of age, seven that are past forty, and twenty-eight that are past thirty: and because it is so frivolous there is only one lawyer, three dentists, and four M.D's in the club, the rest being only common business men. For that reason also, only four of the seventeen representatives of the Ohio Division of the League of American Wheelmen are dentists.

It is also objected that many persons soon give up riding. Yes, that is true. I once knew a young man who was in the habit of taking in such quantities of alcoholic stimulants that his wheel frequently rebelled and pitched him into the middle of the road. He gave up riding because he believed it was injurious to his health. Another rider, who patronized every saloon on the road till his stomach began to shirk its duties, also came to the conclusion that cycling was not good for his health. Then per-persons suffering from gonorrhœa, syphilis and similar infantile diseases have given up riding for the same reason. There is another class that soon give up riding, namely, those who imagine they will be looked upon as novices unless they ride farther or faster than every one else, and, in their desire to emulate the example of trained athletes, become slaves to their wheels and find the sport tiresome and anything but beneficial.

There are some, however, who have not given up riding, who lock up their offices on Sunday morning, and, leaving the aroma of dead nerves, and all thoughts of carious teeth behind them, mount their wheels, and, wandering through the rural scenes, feast their eyes on the beauties of nature. When such persons return to their offices they take with them the songs of birds, the fragrance of flowers, and bits of sunshine to brighten up their lives for the following week.

